# The Effects of Diabetes Induction on the Rat Heart: Differences in Oxidative Stress, Inflammatory Cells, and Fibrosis between Subendocardial and Interstitial Myocardial Areas

**DOI:** 10.1155/2017/5343972

**Published:** 2017-07-11

**Authors:** Maria C. Guido, Alyne F. Marques, Elaine R. Tavares, Marcelo D. Tavares de Melo, Vera M. C. Salemi, Raul C. Maranhão

**Affiliations:** ^1^Laboratory of Metabolism and Lipids, Heart Institute (InCor) of the Medical School of the University of São Paulo, São Paulo, SP, Brazil; ^2^Heart Failure Unit and Clinical Cardiology Division, Heart Institute (InCor) of the Medical School of the University of São Paulo, São Paulo, SP, Brazil; ^3^Faculty of Pharmaceutical Sciences, University of São Paulo, São Paulo, SP, Brazil

## Abstract

Diabetic cardiomyopathy (DCM) is characterized by cardiac remodeling and impaired diastolic function that may lead to heart failure. The aim of this study was to evaluate oxidative stress, inflammatory cells, and fibrosis in both subendocardial (SEN) and interstitial (INT) areas of the myocardium. Male Wistar rats were allocated to 2 groups of 9 animals, a control (CT) group and streptozotocin-induced diabetes (DM). After 8 weeks, echocardiography morphometry, protein expression, and confocal microscopy in SEN and INT areas of the left ventricle (LV) were performed. The echocardiographic analysis showed that diabetes induction leads to cardiac dilation, hypertrophy, and LV diastolic dysfunction. As compared to CT, the induction of diabetes increased inflammatory cells and fibrosis in both SEN and INT areas of DM myocardium and increased ROS generation only in SEN. Comparing the SEN and INT areas in the DM group, inflammatory cells and fibrosis in SEN were greater than in INT. In conclusion, diabetic myocardium SEN area, wherein oxidative stress was more pronounced, is more susceptible to cardiac dysfunction than INT area. This finding can be important for the understanding of the heart remodeling process occurring in DCM and perhaps to engender targeted therapies to attenuate or revert DCM-related diastolic dysfunction.

## 1. Introduction

Diabetic cardiomyopathy (DCM) is characterized by increased cell death, myocyte hypertrophy, myocardial fibrosis, and impaired diastolic function that may lead to heart failure (HF) [1]. Hyperglycemia and abnormal insulin signaling enhance the production of advanced glycation end-products (AGES) and of their receptors (RAGE) in the heart. AGES bind to slow turnover proteins, such as collagen, promoting collagen crosslinking that accounts for the increase of myocardial stiffness and is directly related to DCM diastolic dysfunction [2, 3].

The AGES-RAGE axis also leads to increase in the myocardium of the number of inflammatory cells and of reactive oxygen species (ROS). The oxidative stress contributes to endothelial dysfunction, thus diminishing the blood flow to the myocardium [2, 4]. Cardiac remodeling of the diabetic heart occurs as a result of cellular, structural, and functional unbalance among different myocardial areas [5]. A previous strain echocardiography study of the endocardial, mesocardial, and epicardial areas of myocardium showed that only the function of the endocardial area is impaired in DCM [6]. Myocyte necrosis and left ventricle (LV) fibrosis were increased in the endocardium [4, 7]. Endothelial dysfunction and regional low perfusion have been documented in the endocardial area of the diabetic heart and contribute to HF [8].

Previously, in nondiabetic animal models, it was shown that the subendocardial area was more vulnerable than the interstitial area to cardiac dysfunction, due to differences in blood flow between the two areas [9–11]. However, the relationships between the cardiac dysfunction and the markers of inflammation, oxidative stress, and fibrosis as measured separately in the subendocardial and in the interstitial area were not described in DCM. Due to the high incidence of cardiac complications in patients with diabetes, it is important to investigate whether those differences in the two areas also occur in the diabetic heart. Thus, the aims of this study were to evaluate oxidative stress, inflammation, and fibrosis in both subendocardial and interstitial areas of the myocardium and to assess possible correlations of these parameters with myocardial dysfunction occurring in DCM.

## 2. Methods

### 2.1. Animals

Male Wistar rats weighing 350–400 g were used in this experiment. Animals were maintained on standard rat chow and water ad libitum in rooms with controlled temperature and light cycle.

Eighteen rats were used in the study; animals were randomly allocated to 2 groups, 9 with diabetes (DM), and 9 animals as a control group (CT).

All procedures were performed in accordance with the guidelines of the Brazilian College of Animal Research and conform the NIH guidelines. The study protocol was approved by the Ethics Committee of the University of São Paulo Medical School Hospital (005/14).

### 2.2. Induction of Diabetes Mellitus

Diabetes was induced after 6 hours of fasting. Animals were anesthetized with ketamine chlorhydrate (50 mg/kg) and xilazin (25 mg/kg) intraperitoneally (IP). The induction was performed by a single intravenous injection of steptozotocin (STZ) (Sigma, St. Louis, MO) into the tail vein at the of dose 50 mg/kg, diluted in citrate buffer 0.1 M (pH 4.5). Nondiabetic control rats received an injection of citrate buffer alone.

### 2.3. Transthoracic Echocardiography

Transthoracic echocardiography was performed 8 weeks after DM induction using a Sequoia 512 machine (Siemens, Mountain View, CA) equipped with a 10–13 MHz linear transducer, as previously described [12]. Rats were anesthetized following the same protocol as in the diabetes induction, and the following morphometric and functional parameters were obtained from short-axis view at the level of the papillary muscles by M-mode echocardiography [13]. LV mass was calculated by using the following formula: LV mass = 0.8 × {1.04[(LVID + PWTh + IVSTh)^3^ − LVID^3^]} + 0.6.

The sequence of echocardiographic examination was M mode, 2-dimensional color Doppler, and pulsed-wave Doppler of which the variables were measured from the aortic valve closure to the onset of mitral inflow and LV outflow velocity measured just below the aortic valve. The average of 3 consecutive cardiac cycles was used in all echocardiographic parameters.

### 2.4. Blood Biochemistry

Promptly after the final echocardiography study, the animals were euthanized using an overdose (100 mg/kg) of sodium thiopental (Cristália, Itapira, Brazil). Blood samples were taken from the superior vena cava for determination of glycemia and insulin levels. The analyses were performed using a COBAS c111 (Roche, Basileia, Switzerland) and a multiplex immunoassay through MAP kit (MILLIPLEX®, MERK, Darmstadt, Germany), respectively.

### 2.5. Morphometry

A slice of the heart was obtained at the equatorial plane and cut into 5 *μ*m sections. Tissue sections stained with hematoxylin and eosin (HE), and Masson's trichrome underwent morphometric studies using an image analysis system (Leica Q500 iW; Leica Imaging Systems, Cambridge, UK). Two areas of the LV were analyzed separately: subendocardium (SEN) and interstitium (INT). The SEN area was defined as the inner third of LV area and the INT area as the remaining outer two-thirds [9, 14].

Inflammatory cells were counted in HE-stained sections under 400x magnification. The cells were identified according to nuclear and cytoplasmic morphological aspects.

Myocyte necrosis was identified in HE-stained sections under 400x magnification by nuclear pyknosis and karyolysis as well as cytoplasmatic changes including vacuolization, contraction bands, and hypereosinophilia.

To measure myocardial fibrosis, collagen volume fraction was determined in Masson's-stained sections under 200x magnification. The fibrosis was calculated as the percentage of blue-stained connective tissue areas per total myocardium area.

### 2.6. Western Blot Analysis

LV was homogenized in RIPA lysis buffer (Thermo Fisher Scientific, Waltham, MA). The proteins were size-fractionated on polyacrylamide/SDS gel; the separated proteins were then electrophoretically transferred to a nitrocellulose membrane. The membranes were blocked with 5% nonfat milk. After the primary antibodies (Table 1) were incubated overnight, the blots were washed and incubated with horseradish peroxidase-conjugated secondary antibodies (Calbiochem, San Diego, CA). Bands were visualized using enhanced chemiluminescence (Amersham, GE, Fairfield, CT), exposed, and analyzed by an image analyzer (Amersham Imager 600, GE, Fairfield, CT). Values were normalized for expression of GAPDH, and results are expressed as percentage of CT group.

### 2.7. In Situ Reactive Oxygen Species Generation

In situ reactive oxygen species microfluorotopography of the LV was performed with dihydroethidium (DHE, Invitrogen, Carlsbad, CA). LV paraffin sections were deparaffinized and incubated in PBS Tween 1% for 30 minutes at room temperature. Sections were incubated with 5 *μ*M DHE for 60 minutes at 37°C. Images were detected in a Zeiss Axiovert 100M scanning confocal microscope and Axiovision software (Carl Zeiss, Jena, Germany). Parallel reading of images was performed with identical laser acquisition settings. Quantitative analysis of fluorescent images of SEN and INT areas was performed with an image analysis system (Leica Q500 iW; Leica Imaging Systems, Cambridge, UK) under 400x magnification [15].

### 2.8. Statistical Analysis

Data are expressed as means ± SEM. Data were analyzed using the appropriate Student *t*-test or one-way ANOVA complemented by Bonferroni's posttest. Pearson's correlation was used to test potential correlations between E/A wave and inflammatory cells, ROS generation, and myocardial fibrosis in SEN and INT areas. In all analyses, *p* < 0.05 was considered statistically significant. Statistical analyses were carried out using GraphPad Prism v.5 statistical software (GraphPad Software Inc., La Jolla, CA). The data collection and the analysis of the echocardiographic study were performed by a single examiner (V.M.S.) that was blinded to the animal groups.

## 3. Results

### 3.1. Body Weight, Glycemia, Serum Insulin, and RAGE Expression

Initial body weight did not differ between DM and CT (360 ± 12 and 346 ± 12 g, resp.), but the final body weight of DM rats was lower than CT animals (348 ± 12 and 510 ± 16 g, resp., *p* < 0.001).

**Figure 1:**
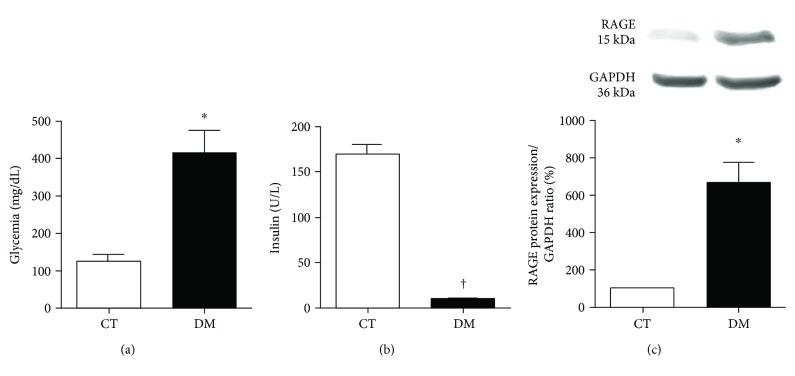
Glycemia (a) and insulin (b) serum concentrations and RAGE protein expression (c) after 8 weeks of diabetes induction. ^∗^*p* < 0.05 and ^†^*p* < 0.001 versus the CT group. Data expressed in mean ± SEM in all plots.

Figure 1 shows the concentration of blood glucose, serum insulin levels, and RAGE expression by Western blot as determined 8 weeks after diabetes induction. Glycemia (Figure 1(a)) was higher in DM compared to CT (*p* < 0.001), whereas serum insulin levels (Figure 1(b)) were lower in DM (*p* < 0.01), confirming the effectiveness of the diabetes induction procedure. RAGE expression was higher in DM than in the CT (*p* < 0.05) (Figure 1(c)).

### 3.2. Transthoracic Echocardiography

As acquired by transthoracic echocardiography performed 8 weeks after the commencement of the experiments, the systolic and diastolic diameters, interventricular septum, LV posterior wall thickness (*p* < 0.01), and E/A wave ratio (*p* < 0.05) were greater in DM than in the CT animals. The isovolumetric relaxation time was lower in DM (*p* < 0.05). Diastolic and systolic volumes, LV mass, deceleration time of E wave, and ejection and shortening fraction were not different between DM and CT (Table 2).

### 3.3. Oxidative Stress

**Figure 2:**
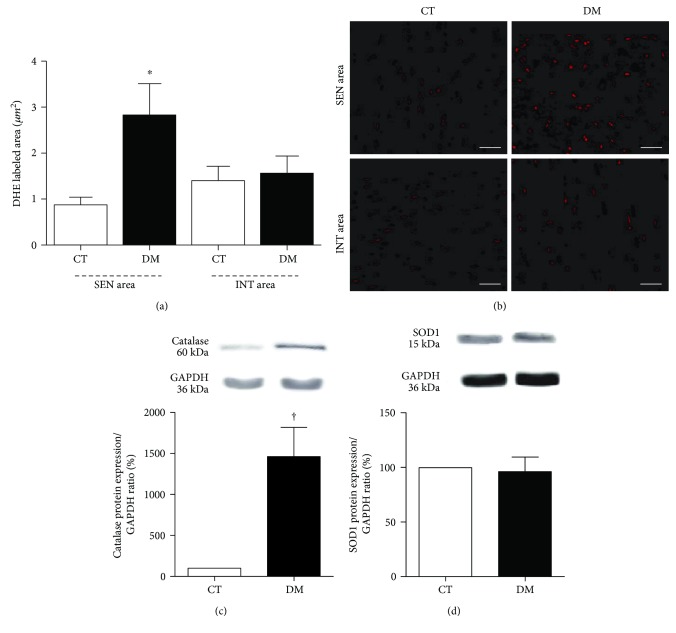
(a) Quantitative analysis of microfluorotopography of DHE oxidation products performed 8 weeks after diabetes induction. ^∗^*p* < 0.01 versus the CT SEN area. Data expressed in mean ± SEM. (b) Representative photomicrograph of SEN and INT areas, showing microfluorotopography of DHE oxidation products. Red staining indicates the fluorescence signal by DHE under 400x magnification. Bars: 50 *μ*m. Western blot protein expression analysis depicting antioxidant enzymes catalase (c) and SOD1 (d) performed 8 weeks after diabetes induction. ^†^*p* < 0.001 versus the CT group. Data expressed in mean ± SEM.

Figure 2 depicts the labeled area of superoxide in the diabetic myocardium. ROS generation was higher in the SEN area of DM (*p* < 0.05) compared to the SEN area of CT (Figure 2(a)).  Figure 2(b) shows the images of superoxide labeled tissue acquired by in situ microfluorotopography from confocal microscopy. Sections incubated with PEG-SOD showed no reaction, confirming that the signal obtained with DHE incubation was specifically from superoxide.

Protein expression of the antioxidant enzyme catalase and SOD1 is shown in Figures 2(c) and 2(d). DM had a higher catalase expression compared to CT (*p* < 0.001) (Figure 2(c)). The SOD1 expression was similar in both animal groups (Figure 2(d)).

### 3.4. Inflammation

**Figure 3:**
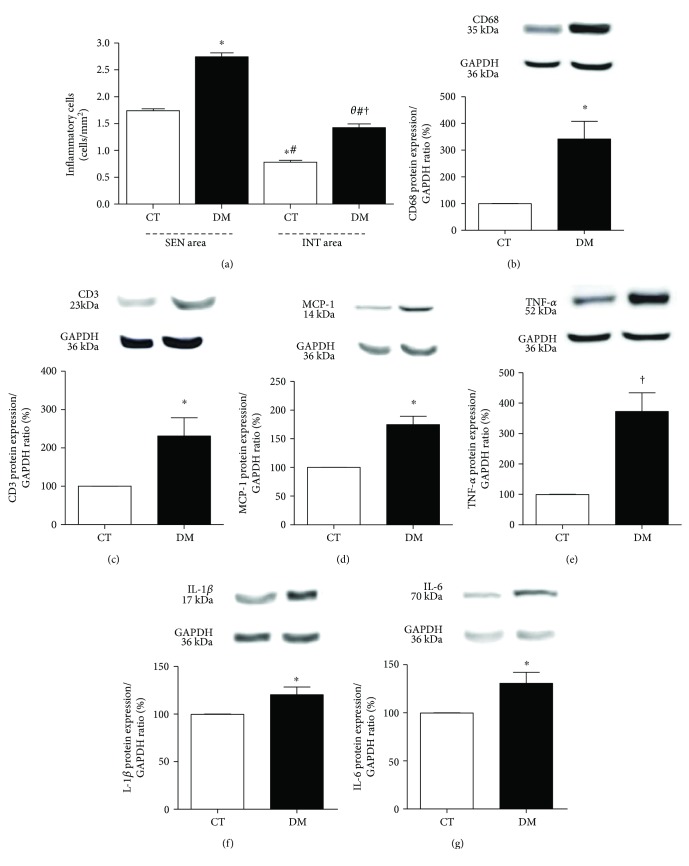
(a) Total inflammatory cell count in SEN and INT areas. HE-stained sections, under 400x magnification. ^θ^*p* < 0.05 versus the DM SEN area; ^∗^*p* < 0.01 versus the CT SEN area, ^#^*p* < 0.01 versus the DM SEN area; ^†^*p* < 0.01 versus the CT INT area. Data expressed in mean ± SEM. Western blot protein expression analysis depicting CD68 (b), CD3 (c), MCP-1 (d), TNF-*α* (e), IL1-*β* (f), and IL-6 (g) was performed 8 weeks after diabetes induction. ^∗^*p* < 0.05 and ^†^*p* < 0.001 versus the CT group. Data expressed in mean ± SEM in all plots.

Figure 3(a) shows the number of inflammatory cells quantified from photomicrographs. In CT, the SEN area showed a higher number of inflammatory cells than the INT area (*p* < 0.001). SEN and INT areas of DM showed a higher number of inflammatory cells when compared to SEN and INT areas of CT, respectively (*p* < 0.001). Interestingly, the DM SEN area showed higher inflammatory cell number than the DM INT area (*p* < 0.001).

DM animals showed a higher expression of CD68 (*p* < 0.05, Figure 3(b)), CD3 (*p* < 0.05, Figure 3(c)), MCP-1 (*p* < 0.05, Figure 3(d)), TNF-*α* (*p* < 0.01, Figure 3(e)), IL-1*β* (*p* < 0.05, Figure 3(f)), and IL-6 (*p* < 0.05, Figure 3(g)) compared to CT.

### 3.5. Cell Death and Hypoxia

**Figure 4:**
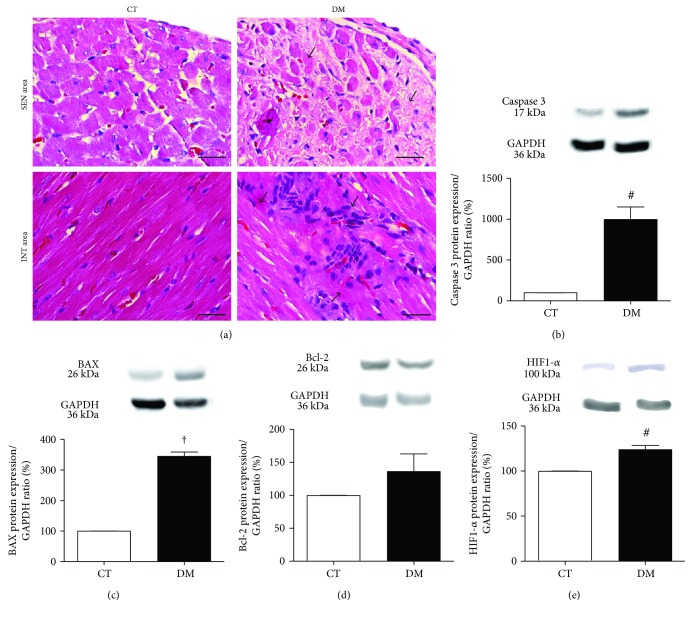
(a) Representative photomicrograph of myocardial necrosis in SEN and INT areas. HE-stained sections under 400x magnification. Arrows indicate nuclear pyknosis, karyolysis, cytoplasmatic changes, and hypereosinophilia. Bars: 50 *μ*m. Western blot protein expression analysis depicting proapoptotic caspase 3 (b) and BAX (c) and antiapoptotic Bcl-2 (d) and HIF1-*α* (e) performed 8 weeks after diabetes induction. ^#^*p* < 0.01 and ^†^*p* < 0.001 versus the CT group. Data expressed in mean ± SEM.

Figure 4(a) shows cell death by necrosis as observed in typical microscopic images in SEN and INT areas of LV myocardium. In DM, the presence of necrotic myocytes in SEN and INT areas was greater compared to CT.

Figures 4(b), 4(c), and 4(d) show the protein expression of the proapoptotic factors caspase 3 and BAX and antiapoptotic factor Bcl-2 by Western blot. Compared with CT, the expression of caspase 3 (Figure 4(b)) and BAX (Figure 4(c)) was higher in DM (*p* < 0.01 and *p* < 0.001, resp.), and the expression of the antiapoptotic factor Bcl-2 was not different between groups (Figure 4(d)). Regarding the protein expression of the HIF-1*α* (hypoxia factor-1*α*), DM was higher in comparison with CT (*p* < 0.01) (Figure 4(e)).

### 3.6. Fibrosis

**Figure 5:**
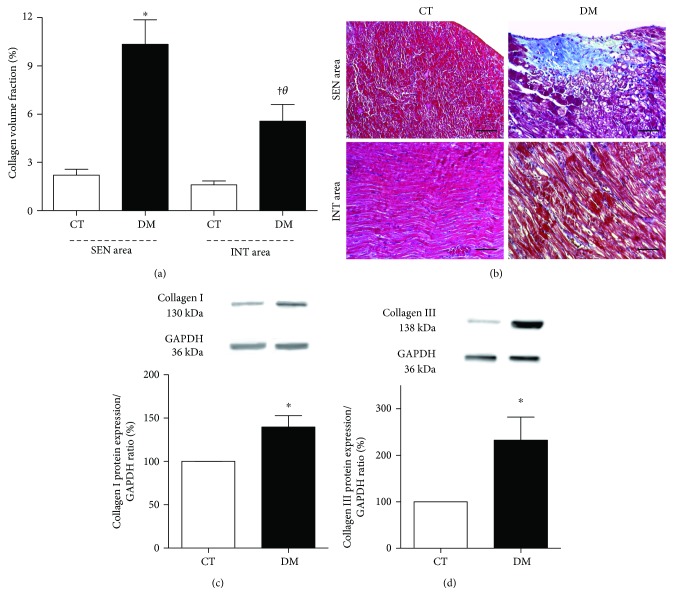
(a) Quantitative analysis of collagen volume fraction in SEN and INT areas, performed 8 weeks after diabetes induction. ^θ^*p* < 0.05 versus the DM SEN area; ^∗^*p* < 0.01 versus the CT SEN area; ^†^*p* < 0.01 versus the CT INT area. Data expressed in mean ± SEM. (b) Representative photomicrograph of SEN and INT areas, showing myocardial fibrosis in Masson's trichrome stain. Blue staining indicates the myocardial fibrosis under 200x magnification. Bars: 100 *μ*m. Western blot protein expression analysis depicting collagen I (c) and collagen III (d), performed 8 weeks after diabetes induction. ^∗^*p* < 0.05 versus the CT group. Data expressed in mean ± SEM in all plots.

The collagen volume fraction (Figure 5(a)) in the SEN area of DM was greater than in the DM INT area (*p* < 0.05), showing a twofold increase. Fibrosis in the SEN area of DM was also greater in comparison to the SEN area of CT (*p* < 0.01), as occurred in the INT area of DM versus CT animals (*p* < 0.05).

Figure 5(b) shows the microscopic images of typical findings of LV myocardial fibrosis. In the DM, the expression of both type I (Figure 5(c)) and type III (Figure 5(d)) collagens in LV was higher (*p* < 0.05) compared with CT.

### 3.7. Correlation between ROS Generation, Inflammatory Cells and Myocardial Fibrosis, and LV Diastolic Dysfunction

To determine the parameters that may be involved in the development of diastolic dysfunction (E/A wave) on DCM, Pearson's correlation was carried out with ROS generation, inflammatory cells, and myocardial fibrosis.

**Figure 6:**
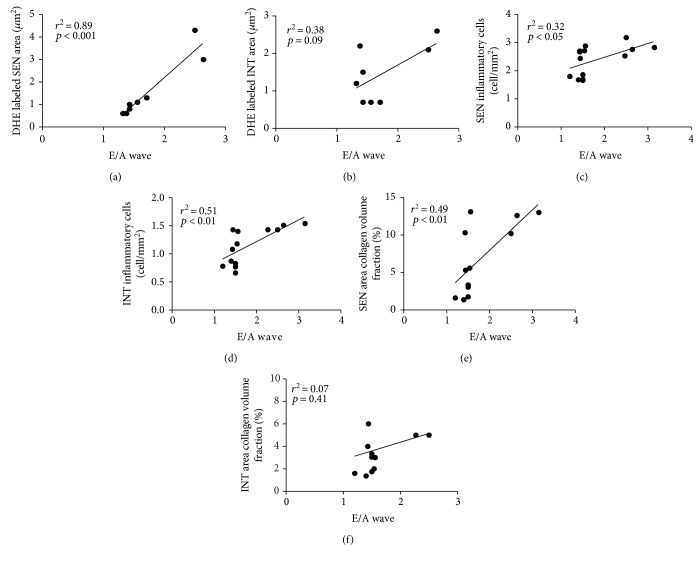
Pearson's correlation between ROS generation (a, b), inflammatory cells (c, d), myocardial fibrosis (e, f) in SEN and INT areas, respectively, versus E/A wave, performed 8 weeks after diabetes induction.

ROS generation in the SEN area correlated positively with diastolic dysfunction (*r*^2^ = 0.89, *p* < 0.001, Figure 6(a)), but this correlation was not present in the INT area (*r*^2^ = 0.38, *p* = 0.09, Figure 6(b)).

E/A wave correlation with total inflammatory cell count was positive in both SEN (*r*^2^ = 0.32, *p* < 0.05, Figure 6(c)) and INT (*r*^2^ = 0.51, *p* < 0.01, Figure 6(d)) areas.

Collagen volume fraction correlated positively with diastolic dysfunction, but only in the SEN area (*r*^2^ = 0.49, *p* < 0.01, Figure 6(e)). In the INT area, there was no correlation (*r*^2^ = 0.07, *p* = 0.41, Figure 6(f)).

## 4. Discussion

In this study, as compared to CT, the induction of diabetes increased inflammatory cells and fibrosis in both SEN and INT areas of DM myocardium and increased ROS generation only in SEN. Comparing the SEN and INT areas in DM group, inflammatory cells and fibrosis in SEN were greater than in INT. The echocardiographic analysis showed that diabetes induction leads to cardiac dilation, hypertrophy, and LV diastolic dysfunction, confirming previous observations in the literature [1, 5–7].

Hyperglycemia and impaired insulin secretion are the main cause of enhanced production of AGES [16]. The increase in AGES generation by diabetes induction was indirectly shown here by the higher expression of RAGE in the DM group. The overexpression of RAGE is strongly connected to myocardial ischemic injury, vascular wall stiffness, and diastolic dysfunction which occur in animals with induced diabetes [17].

The binding of AGES to RAGE increases ROS generation in DCM myocardium, resulting in endothelium dysfunction that contributes to alterations in the microcirculation and oxidative status of diabetic rats [18]. A higher in situ generation of ROS was observed here in the SEN area of DM and was associated with LV diastolic dysfunction. Probably the increase of hypoxia and RAGE contributed to the higher ROS generation in the SEN area, since it was not observed in the INT area of the DM group.

After 8 weeks following diabetes induction, the expression of SOD1 was unchanged; however, the catalase expression was higher compared to CT. Some studies showed that overexpression of antioxidant enzymes is associated with a significant reduction in apoptosis and ROS generation [19, 20]. However, the hyperglycemic state impairs important cofactors that influence the antioxidant defenses [21]. Therefore, the higher catalase expression may not have been sufficient to control ROS generation in the SEN area of the DM group.

DCM increases microvascular permeability, impaired blood flow, ischemia, and subsequent cell death [22]. In a study with diabetic and nondiabetic patients with hibernating myocardium, Mizuno et al. [23] showed that, after complete revascularization, the subendocardial perfusion improved only in nondiabetic patients, which contributed to persistent HF. In addition, other studies showed that the SEN area is more vulnerable than the INT area to effects of hypoxia and ischemia and are directly correlated to cardiac dysfunction [24]. In our study, DM was more prone to cell death, probably due to hypoxia, as showed by the higher expression of HIF1-*α* in the DM group.

The inflammatory process that occurs in DCM is crucial for the adaptive anatomical and functional changes of the heart. ROS upregulation contributes to increase proinflammatory cytokines, chemokines, and activation of macrophages [25]. Some studies showed that the increase of IL-1*β* and IL-6 in DCM is associated with the impairment of the cardiac function [26–29]. Lymphocytes and macrophages infiltrated in the myocardium tissue increased cytokine expression and reduced the insulin signaling [30, 31]. Here, the expression of all cytokines was higher in DM, especially TNF-*α* expression that was about fourfold higher. MCP-1 expression also increased, suggesting macrophage recruitment to the myocardium. We showed for the first time the number of inflammatory cells distributed in SEN and INT areas of DCM. Both SEN and INT areas of the DM group showed a higher number of inflammatory cells compared to CT, and this increase in both myocardial areas was associated with LV diastolic dysfunction. Interestingly, in the DM group, the SEN area presented twofold higher number of cells when compared to the INT area.

Myocardial fibrosis leads to impairment of LV relaxation, which subsequently compromises the efficiency of LV contraction [32]. The differences in the distribution of fibrotic tissues between SEN and INT areas of DCM have been poorly investigated so far [7]. Our data demonstrated that SEN and INT areas of the DM group had a higher collagen content, mainly type I and III collagens, compared to CT. Noteworthy is the fact that the SEN area displayed twofold greater fibrosis compared to the INT area of DM. The fibrosis in the SEN area showed association with LV diastolic dysfunction. The SEN area is the myocardial layer most affected by the perfusion deficits that trigger the fibrotic reparative process [33]. On the other hand, the fibrosis of the INT area has been ascribed to the presence of inflammatory cells and mediators, such as cytokines, and to the changes in neurohumoral system [30, 34].

In conclusion, the SEN area of the diabetic myocardium is more susceptible to cardiac dysfunction than the INT area. The oxidative stress effects, inflammation, and fibrosis were more pronounced in the SEN area. This finding can be important for the understanding of the heart remodeling process occurring in DCM and perhaps to engender targeted therapies to attenuate or revert DCM-related diastolic dysfunction.

## Figures and Tables

**Table 1:** Primary antibodies used in Western blot technique.

Primary antibody	Supplier	Catalog number	Dilution
Anti-BAX	Abcam	ab7977	1 : 500
Anti-Bcl-2	Abcam	ab59348	1 : 1000
Anti-caspase 3	Abcam	ab2302	1 : 1000
Anti-catalase	Abcam	ab16731	1 : 1000
Anti-CD3	Abcam	ab5690	1 : 1000
Anti-CD68	Abcam	ab125212	1 : 1000
Anti-MCP-1	Abcam	ab25124	1 : 1000
Anti-TNF-alpha	Abcam	ab1793	1 : 1000
Anti-IL-1-beta	Abcam	ab82558	1 : 1000
Anti-IL-6	Abcam	ab83339	1 : 1000
Anti-collagen I	Abcam	ab90395	1 : 1000
Anti-collagen III	Abcam	ab7778	1 : 1000
Anti-GAPDH	Calbiochem	BC1001	1 : 10,000
Anti-HIF1-alpha	Abcam	ab463	1 : 1000
Anti-SOD1	Abcam	ab13498	1 : 1000

**Table 2:** Transthoracic echocardiography performed after 8 weeks in CT and DM groups.

	CT	DM
	(*n* = 9)	(*n* = 9)
LVID (mm/mg)	17.7 ± 0.4	23.4 ± 0.6^†^
LVIS (mm/mg)	11.0 ± 0.3	14.2 ± 0.4^†^
LVEDV (mL/mg)	1.2 ± 0.06	1.4 ± 0.12
LVESV (mL/mg)	1.24 ± 0.06	1.43 ± 0.12
ISVTh (mm/mg)	3.2 ± 0.2	4.3 ± 0.3^#^
PWTh (mm/mg)	3.1 ± 0.2	4.3 ± 0.2^#^
RWTh (mm/mg)	0.36 ± 0.02	0.37 ± 0.03
LV mass (mg/mg^2^)	17.5 ± 0.9	20.2 ± 1.2
E/A waves	1.4 ± 0.1	1.9 ± 0.2∗
IVRT (mseg)	39.2 ± 1.7	34.1 ± 2.5∗
DT (mseg)	40.8 ± 3.6	50.3 ± 5.0
EF (%)	73.3 ± 1.1	74.5 ± 1.8
SF (%)	38.0 ± 1.2	39.0 ± 2.0

LVID: left ventricular end-diastolic internal dimension; LVIS: left ventricular end-systolic internal dimension; LVEDV: left ventricular end-diastolic volume; LVESD: left ventricular end-systolic volume; IVSTh: interventricular septum thickness; PWTh: posterior wall thickness; RWTh: relative wall thickness; DT: deceleration time of E wave; IVRT: isovolumetric relaxation time; EF: ejection fraction; SF: shortening fraction. ^∗^*p* < 0.05; ^#^*p* < 0.01; ^†^*p* < 0.001 versus CT.

## Abbreviations

AGES: Advanced glycation end-products
DCM: Diabetic cardiomyopathy
DHE: Dihydroethidium
EF: Ejection fraction
GAPDH: Glyceraldehyde 3-phosphate dehydrogenase
HE: Hematoxylin and eosin
HF: Heart failure
IL-1*β*: Interleukin 1*β*
IL-6: Interleukin 6
INT: Interstitium
IVSTh: Interventricular septum thickness
LV: Left ventricle
LVID: Left ventricular end-diastolic internal dimension
LVIS: Left ventricular end-systolic internal dimension
MCP-1: Monocyte chemoattractant protein-1
PWTh: Posterior wall thickness
RAGE: Receptor of advanced glycation end-products
ROS: Reactive oxygen species
SEN: Subendocardium
SF: Shortening fraction
TNF-*α*: Tumor necrosis factor-*α*.
